# Special Issue: New Insights into the Pathogenesis and Therapies of IgA Nephropathy

**DOI:** 10.3390/jcm11154378

**Published:** 2022-07-28

**Authors:** Hitoshi Suzuki, Jan Novak

**Affiliations:** 1 Department of Nephrology, Juntendo University Urayasu Hospital, Chiba 279-0021, Japan; 2 Department of Microbiology, University of Alabama at Birmingham, Birmingham, AL 35294, USA

IgA nephropathy (IgAN) is the most common form of primary glomerulonephritis worldwide [1]. Up to 40% of IgAN patients progress to kidney failure within 20 years following diagnosis [2]. Moreover, life expectancy is reduced by a decade in patients with IgAN [3]. IgA vasculitis with nephritis (IgAVN), formerly known as Henoch–Schönlein purpura nephritis, is a systemic form of vasculitis with renal manifestations similar to IgAN and with variable clinical outcome [4]. Thus, IgAN and IgAVN are significant public health problems.

The pathologic assessment of a renal biopsy specimen is the current “gold standard” for diagnosis of IgAN (for review, see [5,6,7,8,9]). Routine immunofluorescence reveals presence of glomerular immunodeposits consisting of IgA, with variable IgG and/or IgM. Complement C3 is present in most cases, but C1q is absent. Light microscopy provides an assessment of disease severity and prognosis based on the Oxford classification system. Its expanded version, termed MEST-C score, provides an evaluation of five different features: Mesangial cellularity, Endothelial hypercellularity, Segmental sclerosis, Tubular atrophy/interstitial fibrosis, and Crescents. IgAVN presents, in addition to kidney vasculitis features, with a greater frequency of severe lesions, such as glomerular necrosis and crescents. However, the pathologic findings for patients with IgAN as well as IgAVN may be impacted by the time between disease onset, diagnostic renal biopsy, and prior medications. Thus, a renal biopsy provides a snap shot in time since the inherent risks associated with renal biopsy hinder the use of a repeat biopsy. Minimally invasive approaches, such as those based on liquid biopsy biomarkers (e.g., blood, urine), are needed for monitoring disease progression, responses to treatments, and the identification of patients at risk of disease progression who may benefit from participation in new clinical trials.

In the last two decades, extensive studies, using genetic, cellular, biochemical, and immunologic approaches, have enabled the formulation of a multi-hit pathogenesis model for IgAN [10], which has been expanded to IgAVN [11]. This hypothesis postulates that glomerular immunodeposits originate from circulating immune complexes consisting of aberrantly glycosylated IgA1 bound by IgG autoantibodies. This IgA1 has some of the 3 to 6 clustered hinge-region *O*-glycans deficient in galactose (galactose-deficient IgA1). These IgA1-IgG circulating immune complexes have been found in patients with IgAN as well as IgAVN. Moreover, glomerular immunodeposits of patients with IgAN are enriched in galactose-deficient IgA1 glycoforms and the corresponding IgG autoantibodies [12,13,14]. Furthermore, the elevated serum levels of galactose-deficient IgA1 and corresponding IgG autoantibodies predict disease progression. The pathogenic role of immune complexes consisting of galactose-deficient IgA1 and IgG autoantibodies was recently confirmed in an experimental animal model [15,16]. Moreover, there are genetic elements involved in disease susceptibility and progression that can be associated with this multi-step mechanism [17,18,19,20,21,22,23].

Despite the progress since the disease was initially described in 1968 [24], disease-specific therapy to slow or prevent the progression of kidney injury is still not available. To develop a curative treatment, new strategies for early diagnosis, disease-specific targets, and methods for the assessment of clinical responses in clinical trials need to be identified and developed.

This Special Issue presents a collection of reviews and clinical and experimental studies focused on specific aspects of the current research of the diagnosis, prognosis, disease pathogenesis, determination of disease activity, and emerging strategies for the treatment of IgAN and IgAVN. Of the twelve papers in this Special Issue, two are focused on IgAVN, nine on IgAN, and one makes a comparison between IgAN and IgAVN. Two papers are related to pathology, specifically, the prognostic significance of crescents in IgAN (Trimarchi et al. [25]) and the light microscopic features observed at different times since disease onset in adult patients with IgAVN (Lai et al. [26]). Another paper compares features of IgAN and IgAVN (Pillebout et al. [27]). Two reviews focus on the two key factors in IgAN, the autoantigen (galactose-deficient IgA1) and the corresponding autoantibodies (Ohyama et al. and Knoppova et al. [28,29]), whereas an experimental study postulates that urinary galactose-deficient IgA1 may be a disease-specific biomarker useful for the assessment of disease activity in IgAN (Fukao et al. [30]). Two additional studies consider the role of complement in disease pathogenesis as it relates to therapeutic approaches or the diagnosis and prognosis of disease progression (Poppelaars et al. and Mizerska-Wasiak et al. [31,32]). Another study postulates that obesity may play a role in mesangial lesions in IgAN (Hong et al. [33]). In the last three papers, the authors review the characteristics of various murine models of IgAN (Wehbi et al. [34]) and provide updates on the current clinical trials in IgAN (Cheung et al. [35] and Maixnerova et al. [36]).

Although the papers in this Special Issue do not cover all current activities in the field, we found these publications to be inspirational and informative. We hope that the readers of this Special Issue will reach the same conclusion.
